# Repurposed Drug Prioritisation Pipeline for an Alzheimer’s Disease Multi‐arm Platform Trial

**DOI:** 10.1002/alz70859_102227

**Published:** 2025-12-25

**Authors:** Sabahat Iqbal, Cristina Bonet Olivares, Laura Rizzo, Thomas D Parker, Charis Wong, Benjamin R Underwood, James Carpenter, Ross Dunne, Vanessa Raymont, Alastair Reith, Catherine J. Mummery, Eric H Karran, Fiona Ducotterd, Gordon Wilcock, Jeffrey L. Cummings, John T O'Brien, Leah Mursaleen, Lon S. S Schneider, Rik Vandenberghe, Suzanne J Reeves, Paresh Malhotra

**Affiliations:** ^1^ Imperial College London, London United Kingdom; ^2^ Division of Psychiatry, University College London, London United Kingdom; ^3^ University of Edinburgh, Edinburgh United Kingdom; ^4^ Department of Psychiatry, University of Cambridge School of Clinical Medicine, Cambridge United Kingdom; ^5^ Cambridgeshire and Peterborough NHS Foundation Trust, Cambridge United Kingdom; ^6^ University College London, London United Kingdom; ^7^ Greater Manchester Mental Health Dementia Research Centre, Manchester United Kingdom; ^8^ Geoffrey Jefferson Brain Research Centre, University of Manchester, Manchester United Kingdom; ^9^ University of Oxford, Oxford, Oxfordshire United Kingdom; ^10^ Dementias Platform UK ‐ University of Oxford, Oxford United Kingdom; ^11^ Breckenfield Consulting Limited, London United Kingdom; ^12^ Dementia Research Centre, University College London, London United Kingdom; ^13^ NIHR UK Dementia Trials Network, University College London, London United Kingdom; ^14^ University of Oxford (Emeritus), Oxford United Kingdom; ^15^ Chambers‐Grundy Center for Transformative Neuroscience, Kirk Kerkorian School of Medicine, University of Nevada, Las Vegas, NV USA; ^16^ Alzheimer's Research UK, Cambridge United Kingdom; ^17^ Alzheimer’s Disease Research Center, Keck School of Medicine, University of Southern California, Los Angeles, CA USA; ^18^ KU Leuven, Laboratory for Cognitive Neurology, Department of Neurosciences, Leuven Brain Institute, Leuven Belgium; ^19^ Imperial College Healthcare NHS Trust, London United Kingdom; ^20^ UK Dementia Research Institute, Care Research and Technology Centre, London United Kingdom

## Abstract

**Background:**

Despite recent developments in amyloid‐clearing therapies for Alzheimer's Disease (AD), there remains a need for more effective, safe treatments. Funded by the UK National Institute of Health and Care Research, our aim was to establish a robust, systematic, and unbiased drug prioritisation pipeline to identify repurposed drugs with the greatest potential for sustained clinical effect in clinical 'real‐world' AD (MMSE >17). These would then be considered for inclusion in a planned multi‐arm UK platform trial.

**Method:**

We invited the wider AD community to propose compounds by summarising their rationale and evidence in a prespecified drug proposal form. Fourteen proposals were received, and the drug proposers were invited to the first panel meeting. They presented a pre‐clinical data focused rationale for each compound to an international expert panel comprised of AD clinicians, scientists, industry professionals, as well as both charity and patient/public representatives. In the context of a platform trial using prespecified primary outcome of cognition (ADAS‐Cog, with neuropsychiatric symptoms and everyday activities as secondary outcomes), the panel ranked their top three compounds based on efficacy, biological plausibility, and safety in AD. Ten compounds were taken forward with two subsequently excluded due to lack of data in humans. We compiled extended drug curriculum vitae (CV) for the eight shortlisted compounds. This incorporated practical clinically relevant information and was supplemented by systematic clinical literature review using Repurposing Living Systematic Review (ReLiSyR), a machine learning tool that searches databases and screens studies to identify relevant publications.

**Result:**

Drug CVs were reviewed in a second panel meeting. Compound rankings were repeated with previous considerations and also practical clinical factors. The highest ranked compounds were atomoxetine (1^st^), metformin (2^nd^), isosorbide mononitrate and levetiracetam (joint 3^rd^). The pivotal determining factors across the top ranked compounds included scientific evidence of plausible mechanistic pathways in AD as well as evidence of substantial clinical data on safety and tolerability.

**Conclusion:**

We present a practical approach to prioritising repurposed drugs to evaluate in the context of clinical AD. We would like to thank our patient and public contributors and the wider investigator team.

